# Elevated factor XI is associated with increased risk of recurrent cerebral venous sinus thrombosis: a cohort study

**DOI:** 10.1007/s11239-023-02935-2

**Published:** 2023-12-25

**Authors:** Elżbieta Paszek, Maciej Polak, Anetta Undas

**Affiliations:** 1 Clinical Department of Interventional Cardiology, John Paul II Hospital, Krakow, 31-202 Poland; 2 Department of Thromboembolic Disorders, Institute of Cardiology, Jagiellonian University Medical College, 80 Pradnicka St, Krakow, 31-202 Poland; 3 Department of Epidemiology and Population Studies, Jagiellonian University Medical College, Krakow, Poland; 4 Krakow Center for Medical Research and Technologies, John Paul II Hospital, Krakow, 31-202 Poland

**Keywords:** Factor VIII, Factor XI, Cerebral venous sinus thrombosis, Thrombosis, Stroke

## Abstract

Cerebral venous sinus thrombosis (CVST) has no identified cause in 15% of cases. Elevated factors (F) VIII and FXI have been associated with thromboembolism, but data on CVST are limited. We hypothesized that elevated plasma FVIII and FXI predispose to first and recurrent CVST. In 50 CVST survivors aged < 60 years, following anticoagulant cessation and in 50 controls, we determined plasma FVIII and FXI, along with fibrin clot properties: lysis time, permeability, maximum D-dimer (D-D_max_), and maximum rate of D-dimer increase (D-D_rate_). We recorded CVST recurrence during a follow-up of 58.5 (55.0–60.0) months. Plasma FVIII was 22.7% higher in CVST than in controls, with elevated FVIII > 150% in 13 (26%) vs. 4 (8%) patients, respectively (p = 0.02). Median FXI tended to be higher in CVST vs. controls (110.5 [99.0-117-0]% vs. 104.5 [97.0-116.0]%, p = 0.07), while FXI > 120% was observed more commonly in the former group (12 [24%] vs. 4 [8%], respectively, p = 0.03). Patients with FVIII > 150% were less likely to achieve complete recanalization compared with the remainder (2 [15.4%] vs. 28 [75.7%], respectively; p < 0.001). Eight patients (16%) experienced CVST recurrence. They had higher baseline FXI, but not FVIII, as compared with the remainder (125.5 [114.5–140.0]% vs. 107.5 [102.0-117.0]%, respectively, p = 0.01). Patients with FXI > 120% were four times more likely to have recurrent CVST (5 [62.5%] vs. 7 [16.7%], respectively; p = 0.01). Plasma FXI > 120% could represent a novel risk factor for first and recurrent CVST. Given advances in anti-FXI agents, CVST might be another indication for this emerging treatment.

## Background and purpose

Cerebral venous sinus thrombosis (CVST) is a rare condition caused by a thrombosis of dural sinuses, with a recurrence risk of 2–7% per year [1–3]. Although several risk factors for CVST such as malignancies, infections, oral contraception, pregnancy, and thrombophilia are well-established, 15% of cases remain cryptogenic [4]. It has been shown that CVST survivors present a persistently unfavorable fibrin clot phenotype, including lower permeability, and hypofibrinolysis [5].

Little is known about the role of coagulation factors (F) in CVST. Several small studies reported elevated FVIII in CVST without conclusive data on its prognostic value [6, 7]. We have shown that FXI is an independent predictor of major cardiovascular events [8] and its impact is related to low fibrin clot permeability and impaired lysability [9]. To our knowledge, there have been no reports on FXI in CVST. Here we investigated whether elevated FVIII and FXI occur in CVST survivors and affect recurrence.

## Methods

### Patients

We studied 50 patients 6–12 months after a first CVST, following anticoagulant discontinuation and 50 healthy controls, matched for age, sex, body mass index (BMI), and family history of venous thromboembolism (VTE). A subgroup of the study population with a shorter follow-up was presented previously [5]. CVST was diagnosed based on computed tomography or magnetic resonance angiography. The exclusion criteria were: age > 60 years, previous thromboembolic events (myocardial infarction, VTE), inflammatory diseases, and malignancy. The study protocol conformed to the ethical guidelines of the 1975 Declaration of Helsinki and was approved by the local ethics committee. Participants provided informed written consent. This study adheres to the STROBE guideline [10].

### Laboratory parameters

Fasting venous blood samples were obtained at one to five months following the end of anticoagulation. Basic laboratory parameters were measured using routine techniques. Fibrinogen was assayed with the von Clauss method. Plasma D-dimer, tissue-type plasminogen activator antigen and plasminogen activator inhibitor-1 (PAI-1) antigen were determined using immunoenzymatic assays (American Diagnostica, Greenwich, CT). Thrombophilia screening was performed as described [11]. Plasma clot permeability was assessed using a pressure-driven system and expressed as a permeation coefficient (Ks), which indicates pore size in fibrin networks [11]. Clot lysis time was assessed using an assay based on a recombinant tissue-type plasminogen activator with tissue factor and phospholipids. The maximum D-dimer levels (D-D_max_) and maximum rate of D-dimer increase (D-D_rate_) during tissue plasminogen activator-induced clot lysis, were measured. Plasma samples were analyzed in duplicates, and the inter- and intra-assay coefficients of variation were < 8%.

FVIII and FXI were measured by one-stage clotting assays using factor-deficient plasma (Siemens, Marburg, Germany), with reference ranges of 70–150% for FVIII and 70–120% for FXI. Elevated factors were defined as values above 150% and 120%, respectively.

Study participants were followed every six months with an on-site or telephone visit. Symptomatic recurrent CVST was confirmed on computed tomography, or magnetic resonance angiography.

### Statistical analysis

Continuous variables were reported as means (standard deviation) or medians (interquartile range), as appropriate. The Kolmogorov–Smirnov test was used to test the normal distribution of variables. Categorical variables were reported as numbers and percentages. The differences in the variables were tested using the Student’s test or the analysis of variance and the χ2 test.

We used a logistic regression model to assess the influence of FVIII > 150% and FXI > 120% on CVST. Results of the model were presented as odds ratio with 95% confidence interval (95% CI). Analyses were performed using IBM Corp. software released in 2021 (IBM SPSS Statistics for Windows; version 28.0.; IBM Corp, Armonk, NY, USA) or R Core Team 2013 (R: A language and environment for statistical computing; R Foundation for Statistical Computing, Vienna, Austria). A p-value < 0.05 was considered statistically significant.

## Results and discussion

**Fig. 1:**
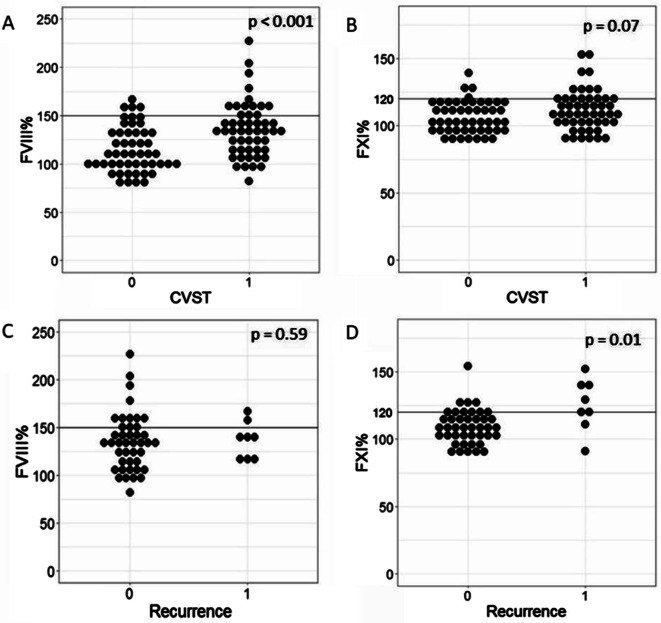
Factor (F)VIII and XI levels with regard to the index cerebral venous sinus thrombosis (CVST) and recurrence. **A** – Factor VIII and CVST; **B** – Factor XI and CVST; **C** – Factor VIII and recurrent CVST; **D** – Factor XI and recurrent CVST

Patients and controls were similar in terms of demographic and laboratory variables. Plasma FVIII was higher in CVST patients than in controls (135.0 [114.0-153.0]% vs. 110.0 [96.0-134.0]%, p < 0.001), and a level above 150% was more frequent in the former group (13 [26%] vs. 4 [8%], respectively, p = 0.02, Fig. 1A). Median FXI tended to be higher in the CVST group compared with controls (110.5 [99.0-117-0]% vs. 104.5 [97.0-116.0]%, p = 0.07), while elevated FXI > 120% was observed much more commonly in the former group (12 [24%] vs. 4 [8%], respectively, p = 0.03, Fig. 1B). FVIII > 150% and FXI > 120% were associated with higher risk of CVST (OR 4.0, [95% CI 1.2–13.6], p = 0.02 and OR 3.6 [95% CI 1.1–12.4], p = 0.04; respectively).

In CVST, patients with FVIII > 150% were younger than the remainder without other differences, including fibrin clot properties (Table 1). Full recanalization at the end of anticoagulation was less frequent at elevated FVIII (Table 1).

**Table 1:** Patient characteristics with regard to plasma FVIII and FXI

	The whole group, n = 50			FVIII ≤ 150%, n = 37			FVIII > 150%, n = 13			P value	FXI ≤ 120%, n = 38			FXI > 120%, n = 12			P value
Age, years	39.5 (31.0–48.0)			42.0 (36.0–48.0)			33.0 (26.0–39.0)			0.03	39.0 (30.0–46.0)			42.5 (35.5–50.5)			0.24
BMI, kg/m^2^	26.5 (24.0–30.0)			27.0 (24.0–30.0)			26.0 (25.0–29.0)			0.78	26.5 (24.0–30.0)			27.0 (24.5–31.0)			0.57
Female, n (%)	36 (72)			26 (70.3)			10 (76.9)			0.47	28 (73.7)			8 (66.7)			0.45
Anticoagulation treatment, months	7.0 (6.0–9.0)			7.0 (6.0–10.0)			7.0 (6.0–9.0)			0.78	7.5 (6.0–9.0)			6.5 (6.0–10.0)			0.98
Complete recanalization, n (%)	30 (60)			28 (75.7)			2 (15.4)			< 0.001	25 (65.8)			5 (41.7)			0.13
**Laboratory parameters**																	
D-dimer, ng/ml	237.0 (197.0-340.0)			233.0 (180.0-319.0)			248.0 (215.0-459.0)			0.22	234.5 (180.0-352.0)			242.0 (206.0-303.5)			0.96
Fibrinogen, g/l	3.0 (2.7–3.3)			3.0 (2.7–3.3)			3.0 (2.7–3.1)			0.63	3.0 (2.7–3.3)			2.9 (2.3–3.1)			0.26
tPA, ng/ml	9.8 (8.4–11.0)			9.8 (8.5–11.2)			9.5 (8.1–10.5)			0.58	9.7 (8.1–11.2)			9.9 (8.9–10.5)			0.96
PAI-1, ng/ml	25.5 (20.4–30.1)			25.2 (21.2–30.7)			26.9 (20.0–29.0)			0.42	25.5 (20.6–30.0)			25.2 (20.1–31.8)			0.84
FVIII, %	135.0 (114.0-153.0)			125.0 (110.0-137.0)			162.0 (158.0-178.0)			< 0.001	136.0 (118.0-153.0)			134.0 (106.5-148.5)			0.46
FXI, %	110.5 (102.0-120.0)			111.0 (103.0-119.0)			105.0 (94.0-120.0)			0.19	106.0 (98.0-113.0)			127.5 (122.0-140.0)			< 0.001
**Fibrin clot properties**																	
Ks, 10^− 9^cm^2^	6.5 (5.9-7.0)			6.5 (5.9-7.0)			6.5 (5.9-7.0)			0.69	6.5 (5.9-7.0)			6.8 (5.7–7.9)			0.29
D-D_rate_, mg/l/min	0.070 (0.065–0.073)			0.070 (0.066–0.074)			0.070 (0.065–0.072)			0.52	0.070 (0.065–0.073)			0.069 (0.066–0.072)			0.49
D-D_max_, mg/l	4.3 (4.1–4.8)			4.3 (4.0-4.9)			4.3 (4.2–4.8)			0.57	4.3 (4.1–4.8)			4.6 (4.1–4.9)			0.45
Clot lysis time, min	103.5 (89.0-115.0)			104.0 (90.0-117.0)			102.0 (85.0-110.0)			0.45	102.5 (88.0-117.0)			104.5 (94.0-112.5)			0.85

Abbreviations: APTT, Activated Partial Thromboplastin Time; BMI, Body Mass Index; D-D_max_, maximum D-dimer levels in lysis assay; D-Drate, rate of D-dimer release; F, factor; Ks, permeability coefficient; PAI-1, plasminogen activator inhibitor-1; tPA, tissue plasminogen activator

## Follow-up

During a median follow-up of 58.5 (55.0–60.0) months, eight patients (16%) experienced recurrent CVST. Baseline characteristics did not differ with regard to recurrence, except for D-D_max_, which was 14% higher in patients with recurrence (Table 2). Patients with and without recurrence did not differ in FVIII levels (Table 2; Fig. 1C), while baseline FXI was 17% higher and 3.7 times more frequently above 120% in patients with CVST recurrence (Table 2; Fig. 1D).

**Table 2:** Patients with and without CVST recurrence in long-term follow-up

Factor	With recurrence (n = 8, 16%)	Without recurrence (n = 42, 84%)	p-value
Age, years	39.5 (35.5–44.5)	39.5 (30.0–48.0)	0.79
Female, n (%)	4 (50.0)	10 (23.8)	0.28
BMI, kg/m^2^	27.0 (25.0–30.0)	26.5 (24.0–30.0)	0.58
**Laboratory parameters**			
Fibrinogen, g/L	3.3 (2.7–3.7)	2.9 (2.7–3.2)	0.16
D-dimer, ng/mL	229 (189–304)	237 (197–352)	0.67
tPA, ng/mL	10.2 (8.9–10.6)	9.7 (8.1–11.3)	0.98
PAI-1, ng/mL	25.6 (20.9–27.1)	25.5 (20.4–30.3)	0.99
FVIII > 150%, n (%)	2 (25.0)	11 (26.2)	1.00
FVIII, %	140.0 (118.0-151.0)	134.5 (111.0-153.0)	0.59
FXI > 120%, n (%)	5 (62.5)	7 (16.7)	0.01
FXI, %	125.5 (114.5–140.0)	107.5 (102.0-117.0)	0.01
FVIII > 150% and FXI > 120%, n (%)	2 (25.0)	1 (2.4)	0.06
**Fibrin clot properties**			
Ks, 10^− 9^ x cm^2^	5.5 (4.9–7.1)	6.5 (6.0–7.0)	0.11
D-D_max_, mg/l	4.9 (4.5–5.3)	4.3 (4.0-4.7)	0.006
D-D_rate_, ml/ml/min	0.063 (0.062–0.071)	0.068 (0.065–0.071)	0.19
Clot lysis time, min	98.0 (85.0-118.5)	104.0 (89.0-113.0)	0.89

Abbreviations: see Table 1

This study is the first to show that elevated plasma FXI is detectable in 24% of CVST survivors and is associated with recurrent CVST in long-term follow-up. Moreover, elevated FVIII predisposes to incomplete recanalization. Vecht et al. showed a 15-fold increase in the risk of CVST at elevated FVIII [6]. In that study median FVIII levels in CVST survivors were higher than ours and more than 80% of CVST survivors had elevated FVIII, as opposed to 26% in our cohort [6]. This difference is probably related to patient characteristics in that study [6] including a shorter interval since CVST, a larger proportion of women and patients with prior thrombosis, cancer, or inflammatory diseases [12]. We expanded the previous report by showing follow-up data and no association between FVIII and CVST recurrence, in contrast to FXI.

Mechanisms behind FXI elevation in CVST are unclear and likely multifactorial including inherited traits. A recent proteomic study found that in both the acute and chronic (12 months post initial diagnosis) phase of VTE, FXI is associated with proteins involved in inflammation, apoptosis, lipid metabolism, extracellular matrix degradation, and protein misfolding [13], although there were no associations between FXI and CRP or lipids.

In line with our former report [5], we demonstrated that D-D_max_, a marker of fibrin clot density, is linked with recurrent CVST. This supports data linking fibrin clot properties with recurrent thrombosis [12].

Considering ongoing trials on anticoagulation, including anti-FXI agents in several indications [14, 15], it is tempting to speculate that use of anti-FXI agents might constitute a therapeutic option for CVST patients.

This study has limitations. The cohort was small, however representative of a CVST population. It cannot be excluded that FVIII and FXI were partly elevated as a consequence of CVST, however this seems unlikely given the shortest time from diagnosis to blood collection was six months.

In conclusion, elevated plasma FXI associated with the risk of CVST recurrence might help identify patients who may benefit from prolonged anticoagulation or FXI inhibitor-based therapy, if available. It might be hypothesized that CVST survivors should be screened for elevated FXI to optimize the long-term management.
